# Effects of rhizoma peanut cultivars (*Arachis glabrata* Benth.) on the soil bacterial diversity and predicted function in nitrogen fixation

**DOI:** 10.1002/ece3.5735

**Published:** 2019-11-05

**Authors:** Xiao‐Bo Wang, Chih‐Ming Hsu, José C. B. Dubeux, Cheryl Mackowiak, Ann Blount, Xing‐Guo Han, Hui‐Ling Liao

**Affiliations:** ^1^ North Florida Research and Education Center University of Florida Quincy FL USA; ^2^ Erguna Forest‐Steppe Ecotone Research Station Institute of Applied Ecology Chinese Academy of Sciences Shenyang China; ^3^ Key Laboratory of Vegetation Ecology Ministry of Education Northeast Normal University Changchun China; ^4^ North Florida Research and Education Center University of Florida Marianna FL USA; ^5^ Institute of Botany Chinese Academy of Sciences Beijing China

**Keywords:** bacterial diversity, biological nitrogen fixation, functional profiles, grasslands, soil microorganisms

## Abstract

There is a growing awareness of the importance of soil microorganisms in agricultural management practices. Currently, much less is known about whether different crop cultivar has an effect on the taxonomic structure and diversity, and specific functions of soil bacterial communities. Here, we examined the changes of the diversity and composition and enzyme‐encoding nitrogenase genes in a long‐term field experiment with seven different rhizoma peanut cultivars in southeastern USA, coupling high‐throughput 16S rRNA gene sequencing and the sequence‐based function prediction with Tax4Fun. Of the 32 phyla detected (Proteobacteria class), 13 were dominant: Acidobacteria, Alphaproteobacteria, Actinobacteria, Betaproteobacteria, Bacteroidetes, Verrucomicrobia, Gammaproteobacteria, Deltaproteobacteria, Gemmatimonadetes, Firmicutes, Nitrospirae, Chloroflexi, and Planctomycetes (relative abundance >1%). We found no evidence that the diversity and composition of bacterial communities were significantly different among different cultivars, but the abundance of some dominant bacterial groups that have N‐fixation potentials (at broad or fine taxonomic level) and predicted abundances of some enzyme‐encoding nitrogenase genes showed significant across‐cultivar differences. The nitrogenase genes were notably abundant in Florigraze and Latitude soils while remarkably lower in Arbook and UF_TITO soils when compared with other cultivars, indicating different nitrogen fixation potentials among different cultivars. The findings also suggest that the abundance of certain bacterial taxa and the specific function bacteria perform in ecosystems can have an inherent association. Our study is helpful to understand how microbiological responses and feedback to different plant genotypes through the variation in structure and function of their communities in the rhizosphere.

## Graphical abstract

There were no significant among‐cultivar differences in diversity and composition but the abundance of some dominant bacterial groups that have N‐fixation potentials and predicted abundances of some enzyme‐encoding nitrogenase genes were different among cultivars.

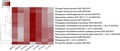

## 1 INTRODUCTION

Rhizosphere microorganisms in cropland systems are critical members of the plant microbiome regulating key aspects of plant growth, nutrient cycling, and soil health. Plant roots can promote or impede rhizosphere microorganisms' recruitment through the secretion of root exudates and volatiles (Berendsen, Pieterse, & Bakker, 2012; Broeckling, Broz, Bergelson, Manter, & Vivanco, 2008; Schmidt, Ulanova, Wick, Bode, & Garbeva, 2019). For example, recent researches have reported that rhizosphere microbial community assembly and microbial taxa colonization can be largely determined by root secondary metabolites of host plants (Veach et al., 2019), including the chemical composition and compounds quality (Gransee & Wittenmayer, 2000; Lesuffleur, Paynel, Bataille, Deunff, & Cliquet, 2007). Root exudates will thus affect the composition and diversity of rhizosphere microbial communities (Aira, Gomez‐Brandon, Lazcano, Baath, & Dominguez, 2010; Badri & Vivanco, 2009), but such an effect may depend greatly upon the plant species and genotype (Rengel & Marschner, 2005). Different plant genotypes are likely to develop distinct microbial communities via the interactions between plant roots and microorganisms, which may also result in the changes of microbial‐driven function in the soil (Philippot, Raaijmakers, Lemanceau, & Putten, 2013; Turner, James, & Poole, 2013). Leveraging on next generation amplicon sequencing protocols, we have highlighted the importance of various biotic, abiotic, and spatial factors in structuring microbial assemblage (Berg & Smalla, 2009; Wang et al., 2017). However, more direct evidence is needed to understand the involvement of genomic‐basis factors in selecting for soil microbiomes. Particularly, the knowledge of cultivar effects (different plant genotypes) on soil microbial communities remains very limited. In agricultural ecosystems, growing evidence has emerged that the composition and diversity of the soil microbial communities differed among cultivars of crops, including wheat (Germida & Siciliano, 2001; Siciliano, Theoret, Freitas, Hucl, & Germida, 1998), potato (Manter, Delgado, Holm, & Stong, 2010; Weinert et al., 2011), sorghum (Schlemper, Veen, & Kuramae, 2018), and rice (Briones et al., 2002; Feng et al., 2015). To date, however, few studies were conducted to evaluate the effects of cultivars in legumes, such as rhizoma peanut (RP, *Arachis glabrata* Benth.) on the soil microbial communities.

Rhizoma peanut is a warm‐season, perennial legume crop that is well‐adapted to the southern United States (Dubeux et al., 2017; Ortega, Sollenberger, Quesenberry, Cornell, & Jones, 1992), which makes it a good option for pasture integration (Garay, Sollenberger, Staples, & Pedreira, 2004; Williams, Hammond, Kunkle, & Spreen, 1991). Rhizoma peanut has a high potential for increasing soil N supply and improving litter quality in C4 grass pastures (Cathey, Sinclair, & Mackowiak, 2013). It can be grown in monoculture for hay or in combination with perennial grasses for grazing systems (Castillo et al., 2015). Similar to many other legume species, RP largely relies on its rhizobium symbionts for biological nitrogen fixation (BNF), which is a primary source of nitrogen (N) in agricultural systems (50–70 Tg N annually, Herridge, Peoples, Boddey, 2008). A recent study has shown that the potentials of BNF in RP are genotype dependent, which is probably attributed to the differences in rhizosphere microbial communities (Dubeux et al., 2017).

Nitrogenase‐mediated BNF process has been reported to benefit legume growth by the improvement of the availability of N that can be directly used by plants (Mus et al., 2016; Welbaum, Sturz, Dong, & Nowak, 2004). BNF, a key component of the nitrogen cycle, is particularly important in agricultural ecosystems because it helps reduce N fertilizer utilization and resulting environmental issues related to N fertilizer inputs such as ammonia volatilization and nitrate leaching (Ju et al., 2009). Bacteria are central performer in BNF process and reveal considerable biodiversity among diazotrophic organisms. It is estimated that 15,000 legume species form symbiotic associations with N‐fixing rhizobia bacteria such as Rhizobia belonging to Alphaproteobacteria (Sprent, 2001). In addition to Rhizobia, N‐fixation ability has been found in other bacterial phylogenetic groups, including green sulfur bacteria, Firmibacteria, Actinobacteria, Cyanobacteria, and other subdivisions of the Proteobacteria (Dixon & Kahn, 2004; Welbaum et al., 2004). Biological nitrogen fixation is an enzymatic reduction process, which is generally controlled by the nitrogenase enzyme that conventionally consists of the iron (Fe) protein and the molybdenum‐iron (MoFe) protein (Zehr & Turner, 2001). The conventional nitrogenase is encoded by a core of *nif* genes including *nifH*, *nifK*, *nifB*, *nifZ*, *nifE*, *nifN*, *nifZ,* and *nifX,* which are all required for nitrogenase synthesis and catalysis (Dos Santos, Fang, Mason, Setubal, & Dixon, 2012; Mus et al., 2016). To our knowledge, little has been known for assessing the changes of nitrogenase genes in symbiotic bacteria associated with different RP cultivars.

The objective of our study is to identity the influences of different RP cultivars on the community structure and predicted function in N fixation of soil bacterial communities. We describe the diversity (16S rRNA genes) for bacteria using next generation amplicon sequencing. Tax4Fun coupled with metabolic references (Kyoto Encyclopedia of Genes and Genomes [KEGG]) was performed to link the phylogenetic and functional diversity of detected bacterial communities (Asshauer, Wemheuer, Daniel, & Meinicke, 2015).

## 2 MATERIALS AND METHODS

### 2.1 Experimental site and sampling strategy

The study is located at the North Florida Research and Education Center (NFREC) in Marianna, FL (30°52′N, 85°11′W). The mean annual precipitation (MAP) during the past 30 years at the experiment site is 1,360 mm, and mean altitude is 35 m a.s.l. The dominant soil types are classified as Red Bay fine sandy loam (fine‐loamy, kaolinitic and thermic Rhodic Kandiudults; Soil Survey Staff, 1999). The experiment was established in September 2010 with planting seven RP cultivars: Latitude, UF Tito, UF Peace, Florigraze, Arbrook, Ecoturf, and Arblick. These cultivars are well‐adapted to the southeastern states of the USA (Dubeux et al., 2017). Planting material was obtained from the UF/IFAS, NFREC RP germplasm collection. The soil was disked and harrowed before planting. Treatments consisting of seven RP cultivars were allocated in a randomized complete block design. Each treatment had four replicates. The size of each plot was 2 m × 3 m, with 2 m alley between plots. During the experimental period, chemical weed control was performed in April 2015. In addition, in June 2014 and in April 2015, we topdressed 56 and 74 kg/ha potassium (K), 29 and 10 kg/ha phosphorus (P), respectively, in soil. More details regarding the experimental management are given by Dubeux Jr et al. (2017).

Sampling work was carried out in April 2017. To decrease the random effect of spatial variations in soil microbial communities, we selected three soil cores (3 cm diameter × 10 cm depth) in each plot (Table S1). Thus, a total of 84 samples were collected in the experimental site. Each soil sample was placed into a sterile plastic bag and put in the icebox immediately. All samples were then transported to the laboratory within 2 hr where they were sieved to 2 mm, thoroughly homogenized, and stored at 80°C for DNA extraction.

### 2.2 DNA extraction, PCR amplification and sequencing

Total genomic DNA was extracted from 0.5 g of well‐mixed soil for each sample using the DNeasy PowerSoil^®^ Kit (QIAGEN) following the manufacturer's instructions. The concentration and quality of DNA were assessed based on the absorbance ratios (*A*_260_/*A*_280_ and *A*_260_/*A*_230_) by a NanoDrop^TM^ One (Thermo Scientific). Extracted DNA samples were preserved at −20°C until use for PCR.

An amplicon survey of the 16S ribosomal RNA (rRNA) genes (primers 515F/806R targeting the V4 region) was performed to provide a higher resolution and more in‐depth analysis of the taxonomic composition and diversity of the bacterial communities (Caporaso et al., 2012). Amplicon libraries were prepared utilizing a frame‐shift tagging system in the primer sequence (Lundberg, Yourstone, Mieczkowski, Jones, & Dangl, 2013) to generate high diversity libraries necessary for MiSeq sequencing. The frame‐shift method has been reported to be able to eliminate the need for Phi X spiking before sequencing (Lundberg et al., 2013). Three PCR amplification steps were performed with a S1000 Thermal Cycler (Bio‐Rad). Reagents and reaction conditions for three PCR amplification steps were shown in Table S2. More details of PCR amplification are described by Chen, Liao, Arnold, Bonito, & Lutzoni (2018). Obtained PCR products were verified on 1% agarose gels and purified using AMPPure XP beads (Beckman Coulter) following the manufacturer's instructions. Sequencing was conducted on an Illumina MiSeq sequencer (v2 250PE) at the Center for Genomic and Computational Biology, Duke University. The data of raw reads have been deposited into the NCBI Sequence Read Archive under accession number SRA063935.

### 2.3 Bioinformatics analysis of sequencing data

Raw sequences with an average quality score >30 were obtained in MiSeq sequencing machine in FASTQ format. The forward and reverse directions for each sample were generated into separated files. First, the files corresponding to the forward and reverse directions of each sample were merged into a single file and added tags for each sequence. The primers of the forward and reverse directions were then trimmed with up to one mismatch allowed and starting position ≤20. Secondly, forward and reverse reads of same sequence with at least 30 bp overlap and <0.25 mismatches were combined as single sequence by using FLASH v1.2.5 (Magoc & Salzberg, 2011). The sequences were then quality trimmed using Btrim (Kong, 2011) with threshold of QC > 30 over 5 bp window size. After extracting the FASTA format from trimmed FASTQ format, we removed the bases after N and trimmed the sequences based on the length. Only sequences longer than 245 bp and shorter than 260 bp were kept. Thereafter, sequences were clustered into operational taxonomic units (OTUs) using the 97% identity threshold with UPARSE (Edgar, 2013) and singleton OTUs (with only one read) were removed. Final OTUs were generated based on the clustering results. Taxonomic assignment was carried out with the Ribosomal Database Project (RDP) classifier against the SILVA SSU rRNA database (Quast et al., 2013). To correct for sampling effort (number of analyzed sequences per sample), we used a randomly selected subset of 1,800 sequences per sample for subsequent community analysis through the resampled OTU table deposited at Dryad (https://doi.org/10.5061/dryad.7034412). There were three samples with very low reads (from the plot Florigraze, Arbrook and Arblick), and thus, a total of 81 samples were used for analysis. The abovementioned steps were performed using an in‐house pipeline that was built on the Galaxy platform at the Research Center for Eco‐Environmental Sciences, Chinese Academy of Sciences (http://mem.rcees.ac.cn:8080/). Functional profiles were predicted based on the 16S rRNA gene sequencing data using Tax4Fun deposited at Dryad (https://doi.org/10.5061/dryad.7034412). Tax4Fun is a novel tool for predicting functional community profiles based upon bacterial 16S sequencing data. Recent studies have shown that Tax4Fun provides a high correlation between functional profiles predicted by 16S sequencing data and whole metagenome sequence data in the soil system (Asshauer et al., 2015; Kaiser et al., 2016). We here mainly focused on the differences of predicted abundances of nitrogenase genes across cultivars. In order to access the functional diversity of BNF across RP cultivars, 13 genes encoding nitrogenases were selected based on Tax4Fun analysis, including Nif‐specific regulatory protein, nitrogenase (EC:1.18.6.1), NifB, nitrogenase molybdenum‐iron protein alpha chain (EC:1.18.6.1), NifE, NifH, nitrogenase molybdenum‐iron protein beta chain (EC:1.18.6.1), NifN, NifT, NifV, nitrogenase‐stabilizing/protective protein, NifX, and NifZ.

### 2.4 Statistical analysis

All statistical analyses were performed in R version 3.5.0 (R Core Team, 2018). We used the OTU richness, Shannon index, Inverse Simpson index, and Chao 1 to estimate alpha diversity of bacterial communities. A one‐way analysis of variance (ANOVA) followed by least significant difference (LSD) test was performed to detect the significant differences among different cultivars. Nonmetric multidimensional scaling (NMDS) ordination was used to assess variations in bacterial community composition across cultivars. NMDS analyses were performed using the MetaMDS function based on dissimilarities calculated using the Bray–Curtis index. A dissimilarity test of the patterns in composition across sites was performed using nonparametric multivariate statistical tests and analysis of similarities (ANOSIM, 9,999 permutations; Clarke, 1993). Both NMDS and ANOSIM were performed with the vegan and ape R package (Oksanen et al., 2013). Hierarchical cluster analysis of the bacterial communities based on the Bray–Curtis dissimilarity matrix was conducted with the hclust function in package vegan, and classification boxes were added using the ordicluster function.

To investigate the differences of the bacterial taxonomic composition across different cultivars, we first chose 13 dominant phyla (class) based on the taxonomic abundance data (relative abundance >1%) to display the differences among cultivars at the phylum levels. We then chose 20 dominant genera based on the taxonomic abundance data (average abundance >10 across all soil samples) and conducted multivariate data analysis with FactoMineR R package. The methods implemented in the package are conceptually similar with classical multivariate data analysis like principal component analysis (PCA) or correspondence analysis, but the graphical outputs including variables factor map and individuals factor map can display clearer distribution of the dominant bacterial taxa across cultivars than using the traditional PCA method.

## 3 RESULTS

### 3.1 Distribution of bacterial taxa and alpha diversity

Across all soil samples, we obtained a total of 446,107 high‐quality sequences with 1,800–23,857 sequences per sample (mean: 5,507). The sequences were then grouped into 8,443 operational taxonomic units (OTUs) with a 97% sequence similarity threshold. Of these sequences, 99.1% was classified at the phylum level. All samples were compared at an equivalent sequencing depth of 1,800 randomly selected 16S rRNA gene amplicons per sample. The dominant phyla across all samples were Acidobacteria (22.83%), Alphaproteobacteria (14.18%), Actinobacteria (10.57%), Betaproteobacteria (6.93%), Bacteroidetes (6.49%), Verrucomicrobia (6.47%), Gammaproteobacteria (3.60%), Deltaproteobacteria (3.54%), Gemmatimonadetes (2.36%), Firmicutes (2.23%), Nitrospirae (1.84%), Chloroflexi (1.33%), and Planctomycetes (1.16%) (relative abundance > 1% across all samples), accounting for more than 69.4% of all sequences analyzed. Other phyla including Armatimonadetes, Thaumarchaeota, Chlamydiae, Cyanobacteria, and Euryarchaeota were also present in all samples with relatively low abundances, and 14 other rare phyla were identified. At genus level, *GP1* was predominant across all samples. Other abundant genera such as *Gp3*, *Spartobacteria*, *Gp4*, *Gp2*, *Gemmatimonas*, *Gaiella*, *Nitrospira*, *Gp6,* and so on were also observed in this study.

**Figure 1:**
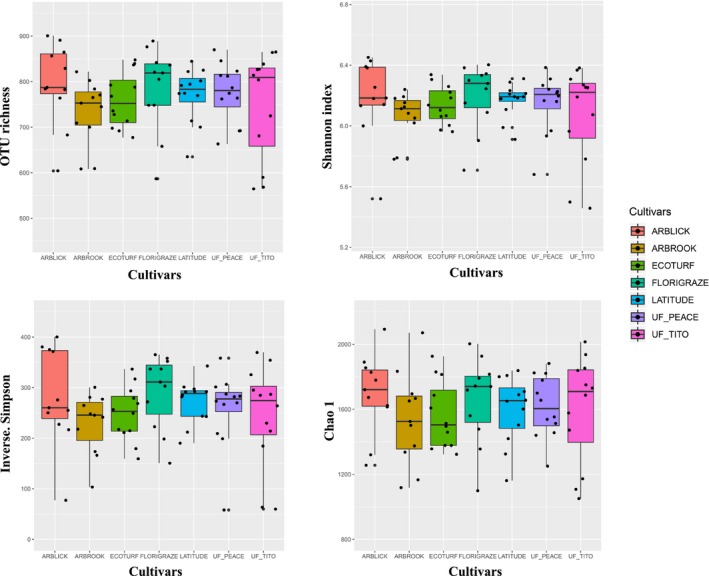
Bacterial alpha diversity estimated by OTU richness, Shannon index, Inverse Simpson, and Chao 1 in soil cropped with seven rhizome peanut cultivars. Points represent the samples in each cultivar. The boxplot shows quartile values for each taxon colored by seven rhizome peanut cultivars. No significant differences were found among different cultivars (One‐way ANOVA, *p* > .05)

The estimated bacterial diversity (i.e., OTU richness, Shannon index, Inverse Simpsons index, and Chao 1) did not vary considerably across seven different cultivars (Figure 1). No statistically significant differences (ANOVA, *p* > .05) in alpha diversity were found for OTU richness, Shannon index, Inverse Simpson index, and Chao 1 under different cultivars (Table 1). Soils from Arblick and Florigraze plots had relatively high diversity while soils from Arbrook plots were relatively low.

**Table 1:** The number of reads and bacterial alpha diversity estimated by OTU richness, Shannon index, Inverse Simpson index, and Chao 1 (mean ± *SE*) using taxon resolution of 97% sequence similarity, respectively in seven different peanut cultivars in Marianna, Florida of USA

Rhizoma peanut	Reads	OTU richness	Shannon index	Inverse Simpson	Chao 1
		*F* = 0.762	*F* = 0.712	*F* = 0.998	*F* = 0.503
		*p* = .602	*p* = .641	*p* = .433	*p* = .805
ARBLICK	5,715	795 ± 27^a^	6.19 ± 0.08^a^	280.95 ± 28.86^a^	1,696 ± 73.40^a^
ARBROOK	4,311	733 ± 22^a^	6.07 ± 0.05^a^	229.09 ± 17.86^a^	1,540 ± 85.75^a^
ECOTURF	5,740	760 ± 17^a^	6.13 ± 0.04^a^	248.64 ± 15.61^a^	1,564 ± 59.68^a^
FLORIGRAZE	7,204	784 ± 28^a^	6.19 ± 0.07^a^	291.58 ± 21.67^a^	1,657 ± 79.58^a^
LATITUDE	5,604	770 ± 17^a^	6.17 ± 0.03^a^	272.84 ± 12.29^a^	1,597 ± 60.03^a^
UF_PEACE	5,474	775 ± 19^a^	6.15 ± 0.06^a^	257.97 ± 21.78^a^	1,618 ± 55.15^a^
UF_TITO	4,564	747 ± 34^a^	6.07 ± 0.09^a^	244.36 ± 29.15^a^	1,599 ± 95.17^a^

Note

Bacterial alpha diversity was calculated with the same surveying effort (*n* = 1,800). Same superscript letters indicate statistical differences at a *p*‐value of >.05 (ANOVA) among sites by least significant difference (LSD) tests.

### 3.2 Bacterial beta diversity

**Figure 2:**
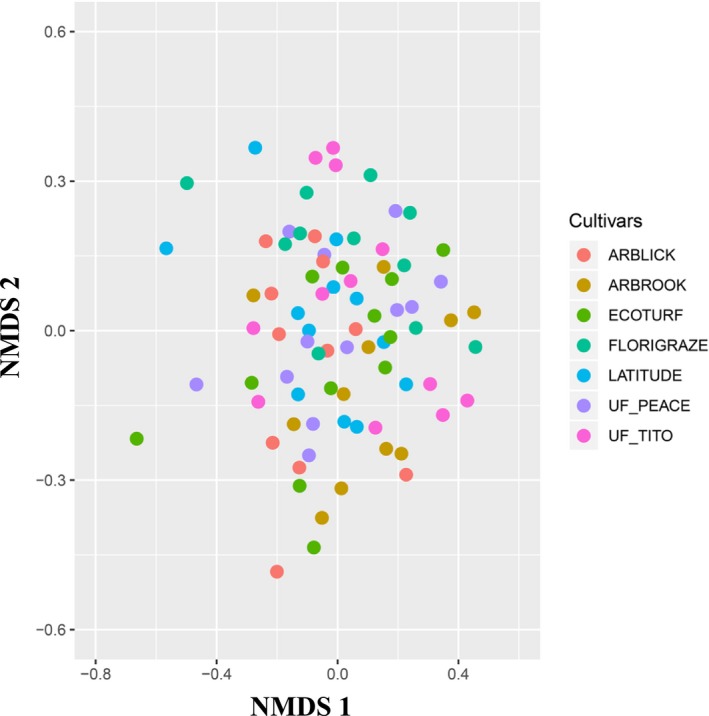
Nonmetric multidimentional scaling (NMDS) ordination with a Bray–Curtis distance matrix of bacterial communities. Points are colored by the different cultivars. There was no significant dissimilarity among different cultivars (ANOSIM)

We further investigated the variation in bacterial beta diversity across different RP cultivars. Community composition similarity across all soil samples was calculated using the Bray–Curtis abundance‐based distance index, which was highly correlated with the incidence‐based Jaccard index (Mantel test: *R* = 0.99 and *p = *.001). The variation in community composition across cultivars is delineated on the first two coordinates of the nonmetric multidimensional scaling ordination. We found no differences in composition among different RP cultivars (Figure 2). The dissimilarity analysis of community composition showed that the bacterial community structures were not significantly different between different cultivars (analysis of similarities: *R* = 0.089 and *p* = .233).

**Figure 3:**
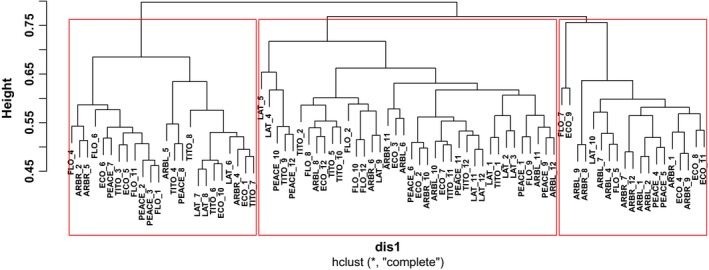
Cluster analysis of bacterial communities based on Bray–Curtis distance matrix. Samples were collected in Marianna in April, 2017. LAT, LATITUDE; ARBL, ARBLICK; ARBR, ARBROOK; TITO, UF_TITO; Peace, UF_PEACE; ECO, ECOTURF; and FLO, FLORIGRAZE. Roman numerals represent the soils which were collected in different replicates. More information was shown in Table S1

Hierarchical cluster analysis showed that the bacterial communities from all soils were clustered into three groups based on Bray–Curtis distance (Figure 3). Group 1 consisted of 19 samples and 14 of which (74%) were from Arblick, Arbrook, and Ecoturf. Group 2 consisted of 43 samples and 31 of which (72%) were from Latitude, UF Tito, UF Peace, Arblick, and Florigraze. Group 3 consisted of 24 samples and 17 of which (71%) were from UF Tito, UF Peace, Florigraze, and Ecoturf.

### 3.3 The taxonomic abundance of bacterial dominant groups

**Figure 4:**
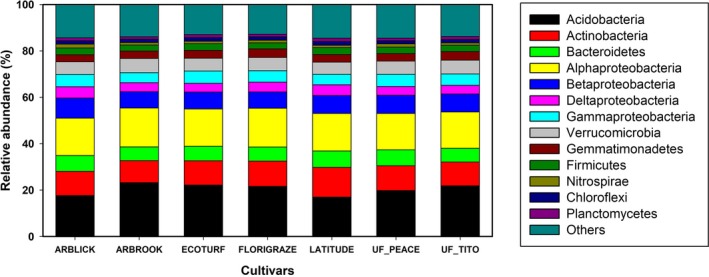
Relative abundance of the dominant groups at the phylum levels (>1% average relative abundance across all soils) of bacterial communities in soil cropped with seven rhizome peanut cultivars

**Figure 5:**
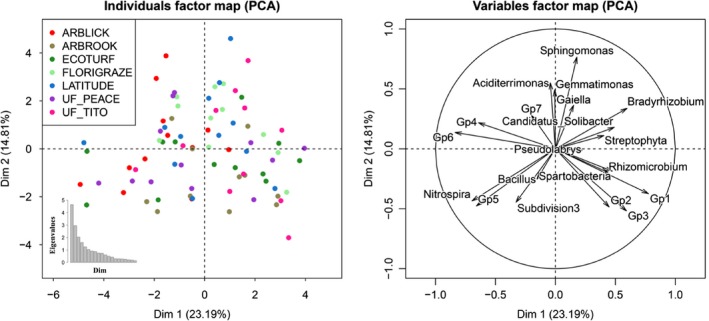
Variables and individuals graph in principal component analysis using PCA function in FactoMineR package. We used taxonomic abundances data (OTUs defined at 97% sequence similarity) from 20 bacterial dominant genera (average abundance >10 across all soil samples) as quantitative variables, which were used to perform the PCA. Individuals were colored from the categorical variables, cultivars. The percentage of variability explained by two dimensions was given: 23.19% for the first axis and 14.81% for the second axis

The relative abundances of dominant bacterial phyla (>1% average relative abundance across all samples) showed some differences across seven cultivars (Figure 4). Significant among‐cultivar differences were found in Alphaproteobacteria, Actinobacteria, Verrucomicrobia, and Nitrospirae (ANOVA, *p* < .05, Table 2). Relative abundance of Alphaproteobacteria in Florigraze soils was significantly higher than that in other RP cultivar soils (LSD, *p* < .05). At a finer taxonomic level, we also found significant differences among RP cultivars in some bacterial genera including *Nitrospira*, *Pseudolabrys*, *Streptophyta*, *Bacillus,* and *Subdivision3_genera_incertae_sedis* (ANOVA, *p* < .05, Table S3). Moreover, the distribution of dominant bacterial genera showed differences across cultivars (Figure 5; Table S3). *Gp4*, *Gp6, and Gp7* were dominant in soils from Arblick. *Gp1*, *Gp3,* and *Subdivision3_genera_incertae_sedis* were dominant in soils from Arbrook. *Sphingomonas*, *Bradyrhizobium,* and *Gaiella* were dominant in soils from Florigraze.

**Table 2:** Relative abundances of dominant bacterial phyla (mean ± *SE*) in soils across different rhizoma peanut cultivars in Marianna, FL, USA

	Rhizome peanut cultivars							
	ARBLICK	ARBROOK	ECOTURF	FLORIGRAZE	LATITUDE	UF_PEACE	UF_TITO	ANOVA (*p*)
Acidobacteria	22.93 ± 1.67 a	24.62 ± 1.56 a	23.51 ± 0.97 a	21.59 ± 1.46 a	20.54 ± 1.08 a	22.19 ± 1.22 a	24.39 ± 2.02 a	.40
Alphaproteobacteria	12.10 ± 0.80 a	12.47 ± 0.73 a	14.70 ± 1.04 b	17.44 ± 1.08 c	14.14 ± 0.40 ab	13.56 ± 0.48 ab	14.72 ± 0.78 b	**.00**
Actinobacteria	9.35 ± 0.65 ab	8.55 ± 0.48 a	9.79 ± 0.70 ab	12.90 ± 0.71 c	11.27 ± 0.77 bc	10.82 ± 0.84 bc	11.26 ± 1.18 bc	**.01**
Betaproteobacteria	7.63 ± 0.66 a	6.38 ± 0.46 a	6.64 ± 0.70 a	5.95 ± 0.18 a	3.53 ± 0.22 a	7.82 ± 0.42 a	6.82 ± 1.20 a	.43
Bacteroidetes	6.74 ± 0.85 ab	5.29 ± 0.59 a	6.06 ± 0.57 ab	6.78 ± 0.78 ab	7.64 ± 0.97 b	6.58 ± 0.80 ab	6.36 ± 0.93 ab	.57
Verrucomicrobia	6.16 ± 0.27 ab	7.32 ± 0.40 c	6.86 ± 0.49 bc	5.35 ± 0.39 a	6.22 ± 0.32 abc	6.85 ± 0.36 bc	6.52 ± 0.47 bc	**.03**
Gammaproteobacteria	3.25 ± 0.35 a	4.52 ± 1.16 a	3.67 ± 0.47 a	3.35 ± 0.28 a	3.59 ± 0.45 a	3.34 ± 0.23 a	3.45 ± 0.30 a	.71
Deltaproteobacteria	4.18 ± 0.23 a	3.54 ± 0.25 ab	3.71 ± 0.26 ac	3.45 ± 0.24 bc	3.53 ± 0.22 ab	3.38 ± 0.22 bc	3.05 ± 0.22 b	.06
Gemmatimonadetes	2.29 ± 0.14 a	2.25 ± 0.18 a	2.15 ± 0.17 a	2.50 ± 0.17 a	2.62 ± 0.32 a	2.36 ± 0.15 a	2.35 ± 0.18 a	.69
Firmicutes	1.91 ± 0.22 a	2.11 ± 0.13 ab	1.94 ± 0.16 a	2.70 ± 0.25 bc	2.74 ± 0.36 bc	2.22 ± 0.27 ab	1.96 ± 0.24 a	.07
Nitrospirae	2.14 ± 0.22 a	1.93 ± 0.24 ab	2.02 ± 0.23 ab	1.24 ± 0.11 c	1.91 ± 0.15 ab	2.13 ± 0.27 a	1.51 ± 0.15 bc	**.02**
Chloroflexi	1.22 ± 0.17 a	1.56 ± 0.11 a	1.30 ± 0.15 a	1.43 ± 0.13 a	1.31 ± 0.21 a	1.16 ± 0.12 a	1.29 ± 0.10 a	.57
Planctomycetes	1.23 ± 0.06 a	1.08 ± 0.10 a	1.26 ± 0.12 a	1.31 ± 0.11 a	1.08 ± 0.07 a	1.09 ± 0.08 a	1.09 ± 0.11 a	.40

Note

Statistics was performed based on the taxonomic abundances data (OTUs defined at 97% sequence similarity) from 13 bacterial dominant phyla (relative abundance >1% across all soil samples). Different letters indicate statistical differences at a *p*‐value of <.05 (ANOVA) among rhizoma peanut cultivars by least significant difference (LSD) tests.

The bold values presented is actually the significance.

### 3.4 Functional profiles of bacterial communities

**Figure 6:**
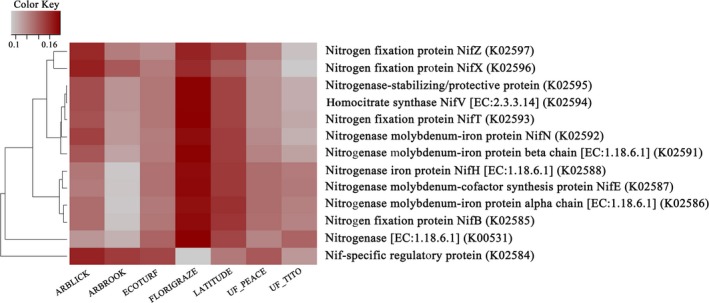
Predicted abundances of enzyme‐encoding genes involved in nitrogen fixation. The color code refers to gene abundance, with high predicted abundances (red) and low predicted abundances (gray)

All nigrogenases genes we examined have been shown functionally associated with bacteria‐driven BNF (Dixon & Kahn, 2004). Generally, the nitrogenase genes were more abundant in Florigraze and Latitude soils while relatively lower in Arbook and UF_TITO soils when compared with other cultivars (Figure 6 and Table 3). The relative abundances of genes for nitrogenase Nif‐specific regulatory protein, nitrogene (EC:1.18.6.1), and nitrogen fixation protein NifT were significantly different among seven rhizoma peanut cultivars (ANOVA, *p* < .05). The relative abundances of genes for Nif‐specific regulatory protein and nitrogenase (EC:1.18.6.1) were extremely high and low, respectively, when associated with cultivars compared to other genes examined. The predicted abundances of genes encoding nitrogenase iron protein (NifH), nitrogenase molybdenum‐cofactor synthesis protein (NifE), nitrogenase molybdenum‐iron protein alpha chain (EC:1.18.6.1), and nitrogen fixation protein (NifB) were relatively lower in Arbrook soils compared with other genes. The predicted abundances of genes encoding Nif‐specific regulatory protein were significantly lower in Florigraze soils compared with the soils grown with Arblick, Arbrook, Ecotorf, and UF Peace (*p* < .05).

**Table 3:** Predicted abundances of enzyme‐encoding nitrogenase genes (mean ± *SE* × 10^–5^) among different rhizoma peanut cultivars in Marianna, Florida, USA using Tax4Fun

	KO number												
	K02584	K00531	K02585	K02586	K02587	K02588	K02591	K02592	K02593	K02594	K02595	K02596	K02597
	*F = *2.70	*F = *2.96	*F = *1.51	*F = *1.30	*F = *1.21	*F = *1.31	*F = *1.26	*F = *2.08	*F = *2.25	*F = *1.27	*F = *2.11	*F = *1.28	*F = *2.09
	*p* = .02	*p* = .01	*p* = .18	*p* = .27	*p* = .31	*p* = .26	*p* = .29	*p* = .07	*p* = .05	*p* = .28	*p* = .06	*p* = .27	*p* = .06
ARBLICK	85.86 ± 3.23 bc	0.16 ± 0.01 ac	7.40 ± 0.22 ab	9.41 ± 0.36 ab	9.16 ± 0.50 a	6.29 ± 0.24 ab	9.87 ± 0.38 ab	5.59 ± 0.18 ab	0.71 ± 0.03 ab	4.67 ± 0.21 a	1.09 ± 0.03 ab	2.02 ± 0.07 ab	1.40 ± 0.04 ab
ARBROOK	83.80 ± 3.38 bd	0.15 ± 0.01 a	6.83 ± 0.25 a	8.77 ± 0.38 ab	8.69 ± 0.49 ab	5.86 ± 0.25 a	9.21 ± 0.40 ab	4.90 ± 0.21 a	0.63 ± 0.03 a	4.32 ± 0.21 ab	0.95 ± 0.04 a	1.87 ± 0.08 ab	1.24 ± 0.05 a
ECOTURF	82.95 ± 2.73 bd	0.18 ± 0.02 ac	7.13 ± 0.47 ab	9.02 ± 0.65 ab	8.42 ± 0.56 ab	6.06 ± 0.44 ab	9.48 ± 0.69 ab	5.51 ± 0.41 ab	0.72 ± 0.05 ac	4.26 ± 0.27 ab	1.07 ± 0.08 ab	1.92 ± 0.13 ab	1.41 ± 0.11 ab
FLORIGRAZE	72.32 ± 4.85 a	0.22 ± 0.02 b	8.00 ± 0.53 bc	10.10 ± 0.72 bc	9.07 ± 0.51 ac	6.81 ± 0.49 bc	10.60 ± 0.75 a	6.35 ± 0.51 b	0.83 ± 0.07 c	4.69 ± 0.26 ac	1.22 ± 0.09 b	2.13 ± 0.13 a	1.61 ± 0.13 b
LATITUDE	78.98 ± 2.44 abd	0.19 ± 0.01 bc	7.58 ± 0.30 ab	9.51 ± 0.38 ab	8.67 ± 0.38 ab	6.38 ± 0.25 ab	9.98 ± 0.40 ab	6.02 ± 0.26 bc	0.78 ± 0.03 bc	4.57 ± 0.21 ab	1.17 ± 0.05 bc	2.04 ± 0.09 ab	1.51 ± 0.06 bc
UF_PEACE	81.49 ± 3.33 bd	0.16 ± 0.01 ac	7.06 ± 0.21 ab	8.80 ± 0.29 ab	8.22 ± 0.28 ab	5.91 ± 0.19 ad	9.25 ± 0.31 ab	5.56 ± 0.19 ab	0.72 ± 0.02 ac	4.30 ± 0.18 ab	1.09 ± 0.04 ab	1.90 ± 0.06 ab	1.42 ± 0.05 ab
UF_TITO	76.50 ± 2.51 ad	0.18 ± 0.01 ac	6.85 ± 0.26 ad	8.55 ± 0.33 ad	7.78 ± 0.27 b	5.76 ± 0.22 ad	8.98 ± 0.34 b	5.40 ± 0.29 ac	0.71 ± 0.04 ab	4.04 ± 0.14 b	1.06 ± 0.05 ac	1.83 ± 0.07 b	1.39 ± 0.07 ac

Note

Different letters indicate statistical differences at a *p*‐value of <.05 (ANOVA) among rhizoma peanut cultivars by least significant difference (LSD) test.

## 4 DISCUSSION

In this study, we investigated the changes of soil bacterial communities with seven different RP cultivars in an experimental field in Florida. We found no significant differences in the taxonomic diversity and composition of bacterial communities among seven RP cultivars (Figures 1, 2, Table 1). The results indicate that the soil with different RP cultivars harbored similar bacterial communities. This finding is not consistent with previous reports showing a significant effect of plant species or cultivar on bacterial community diversity (Marschner, Yang, Lieberei, & Crowley, 2001; Weinert et al., 2010). This is probably because bacterial diversity in soils with rhizoma peanut in our experiment may be primarily determined by the soil properties (Girvan, Bullimore, Pretty, Osborn, & Ball, 2003; M et al., 2001). Although different peanut cultivars were cropped in our field experiment, the soil type, pH, and the conditions of soil moisture and nutrient were similar in the fine‐scale study region. Previous studies have demonstrated that at small or local scales (centimeters to meters), the diversity of soil microbial communities are often determined by variation in the soil physicochemical parameters (Wang et al., 2015; Wardle, 2002). An alternative explanation is that the samples we collected were the mix of rhizosphere and bulk soils. In general, only the root‐associated bacterial communities are relatively sensitive to different cultivars, as host plants and their root exudates have been reported greatly affecting soil bacterial community structures in the previous studies (Marcel, Bardgett, & van Straalen, 2008; Stephan, Meyer, & Schmid, 2000). Furthermore, selection of primers used for amplification of bacterial rRNA gene will affect the outcome of studies examining bacterial community structure. In this study, we only concentrated on bacterial rRNA gene for the bacterial community analysis. However, the relative conservation of the 16S rDNA may not represent all the variation in bacterial community diversity such as certain species‐level variation (Ranjard et al., 2001).

Although no significant differences in diversity and composition of bacterial communities were detected among RP cultivars, taxonomic abundance in some bacterial dominant groups, examined by different taxonomic levels showed significant among‐cultivar differences (Figures 4, 5, Table 2; Table S3). This finding suggests that plant genotypes may not result in a different selection for the specific taxa in a community, but could cause the changes of dominance of certain taxa in that community. Some microorganisms indeed have a particular affinity for certain plant genotypes, which is in accordance with previous reports showing the effects of plant genotypes on microorganisms in the rhizosphere (Bressan et al., 2009; Meyer et al., 2010). Interestingly, significant differences in abundances of dominant bacterial genera (*Pseudolabrys*, *Streptophyta*, *Bacillus*, *Subdivision3_genera_incertae_sedis*, and *Nitrospira*) among RP cultivars were mainly observed for microorganisms belonging to Alphaproteobacteria, Cyanobacteria, Firmicutes, Verrucomicrobia, and Nitrospirae, respectively (Table 2), which were all demonstrated to have the potential ability of N fixation in bacterial communities (Dedysh, Ricke, & Liesack, 2004; DeLuca, Zackrisson, Nilsson, & Sellstedt, 2002; Kalyuzhnaya et al., 2008; Philippot et al., 2013; Reed, Townsend, Cleveland, & Nemergut, 2010; Wertz, Kim, Breznak, Schmidt, & Rodrigues, 2018). Such an among‐cultivar difference of taxonomic abundance of some dominant bacterial groups may suggest a difference of BNF potentials among RP cultivars. In addition, nine dominant phyla including Proteobacteria, Acidobacteria, Actinobacteria, Verrucomicrobia, Bacteroidetes, Gemmatimonadetes, Firmicutes, Nitrospirae, Chloroflexi, and Planctomycetes were detected in our study. This finding is also in agreement with previous reports conducted in peanut cropping soils (Sudini, Liles, Arias, Bowen, & Huettel, 2011).

The availability of N is frequently a major factor affecting crop productivity in agro‐ecosystems. Therefore, understanding BNF potential bacteria is of tremendous importance to agricultural management practices (Vitousek et al., 2002). In our study, we investigated the differences of bacterial functioning in BNF among different RP cultivars, based on predicted abundances of enzyme‐encoding nitrogenase genes using a new bioinformatic tool Tax4Fun. As expected, the predicted abundances of nitrogenase genes showed some differences across RP cultivars (Figure 6, Table 3). This finding is consistent with our predictions, indicating the significant differences of abundance in some BNF‐related taxa. This suggests that plant genome may regulate the metabolic programs that microorganisms use for BNF, or alternatively select the microorganisms that have different BNF efficiency (Berendsen et al., 2012). Higher abundance in most examined nitrogenase genes in Florigraze soils than other cultivars also indicates that Florigraze may have high potentials in BNF.

In conclusion, this study represents an attempt to assess the cultivar effect of RP on soil bacterial communities in a field experiment of southeastern USA. We provided direct evidence that different RP cultivars have no effects on the bacterial taxonomic diversity and composition, whereas the abundance of some dominant bacterial groups that have N‐fixation potentials and the predicted abundances of some nitrogenase genes showed significant among‐cultivar differences. Such a result suggests that bacterial specific function such as nitrogen fixation in ecosystems could be determined by the taxonomic abundance of some dominant groups in a community despite of the characteristics of their community composition and diversity.

## CONFLICT OF INTEREST

The authors declare no conflict of interest.

## AUTHOR CONTRIBUTIONS

H.‐L.L., J.C.B.D., C.‐M., and A.‐B. designed the experiments, X.‐B.W. and C.‐M.H. performed the experiment, X.‐B.W. analyzed the data and wrote the paper with the suggestions of H.‐L.L. and X.‐G. H.. All coauthors contributed to manuscript editing.

## Data Availability

Sequencing raw data has been deposited into the NCBI Sequence Read Archive under accession number SRA063935. The OTU table and predicted functional profiles that were used for taxonomic and functional analysis are available at Dryad (https://doi.org/10.5061/dryad.7034412).
