# Supplementary material for: Effects of rhizoma peanut cultivars (Arachis glabrata Benth.) on the soil bacterial diversity and predicted function in nitrogen fixation
Source: Ecol Evol. 2019 Nov 5;9(22):12676–87. doi: 10.1002/ece3.5735 (PMC6875664; doi:10.1002/ece3.5735)
Supplement: Supplementary file 3 [file ECE3-9-12676-s003.docx]

**Table S3**. The average abundances of the dominant bacterial genera (Mean ± SE) in soils across different rhizoma peanut cultivars in Marianna, Florida, U.S.A. Statistics was performed based on the taxonomic abundances data (OTUs defined at 97% sequence similarity) from 20 bacterial dominant genera (average abundance > 10 across all soil samples).

|  | Rhizome peanut cultivars | | | | | | | |
| --- | --- | --- | --- | --- | --- | --- | --- | --- |
|  | ARBLICK | ARBROOK | ECOTURF | FLORIGRAZE | LATITUDE | UF_PEACE | UF_TITO | ANOVA (*P*) |
| *Gp1* | 89.18±8.72a | 146.64±20.77b | 118.92±16.39ab | 128.00±24.40ab | 90.67±11.24a | 113.25±17.42ab | 116.22±6.50ab | 0.19 |
| *Gp3* | 69.36±5.72a | 88.45±6.93b | 88.33±7.50b | 82.00±5.45ab | 75.67±5.37ab | 75.92±4.51ab | 88.42±7.62b | 0.19 |
| *Spartobacteria_genera_incertae_sedis* | 52.55±3.81a | 59.18±5.13a | 62.25±6.57a | 52.09±5.60a | 50.75±5.65a | 62.83±4.13a | 55.67±5.86a | 0.52 |
| *Gp2* | 49.09±14.61a | 52.45±11.92a | 50.08±8.26a | 32.18±9.29a | 42.50±10.06a | 49.83±19.84a | 62.92±22.89a | 0.88 |
| *Subdivision3_genera_incertae_sedis* | 50.45±2.08ab | 65.36±3.24c | 53.42±4.50a | 35.91±2.18d | 52.75±3.78ab | 53.67±5.50ab | 52.67±3.78ab | **0.00** |
| *Gp4* | 73.64±8.24a | 42.09±6.17b | 54.58±9.60ab | 46.64±7.81b | 52.00±6.89ab | 49.83±8.11b | 47.75±7.66b | 0.16 |
| *Gemmatimonas* | 41.18±2.51a | 40.45±3.25a | 38.75±3.12a | 45.09±2.98a | 47.17±5.77a | 42.50±2.64a | 42.25±3.17a | 0.69 |
| *Gaiella* | 33.91±2.30a | 35.27±3.92ac | 37.83±3.07ab | 46.36±3.05b | 44.33±5.01bc | 41.50±1.94ab | 40.92±3.59ab | 0.12 |
| *Nitrospira* | 38.55±4.02a | 34.73±4.34ab | 36.33±4.13ab | 22.36±1.94c | 34.33±2.76ab | 38.33±4.91a | 27.25±2.76bc | **0.02** |
| *Sphingomonas* | 24.27±3.66a | 20.27±2.51a | 24.67±3.69a | 38.27±4.80b | 29.00±4.03ab | 23.25±2.34a | 30.58±5.68ab | 0.06 |
| *Gp6* | 40.36±4.41a | 24.18±3.31b | 32.50±5.67ab | 25.36±3.29b | 32.67±7.07ab | 33.58±4.43ab | 25.33±3.40b | 0.20 |
| *Bradyrhizobium* | 17.09±2.06a | 17.09±8.50a | 20.42±9.23ab | 26.18±2.39b | 24.50±2.24ab | 24.17±3.77ab | 24.42±3.10ab | 0.11 |
| *Gp7* | 27.09±5.97a | 18.45±2.04ab | 16.58±2.07b | 17.36±2.63b | 18.17±2.20ab | 16.50±2.71b | 22.75±3.15ab | 0.19 |
| *Pseudolabrys* | 12.73±1.21a | 13.64±1.89ab | 15.42±1.39b | 24.45±2.76b | 16.17±1.28ab | 17.17±2.12b | 19.25±1.70ab | **0.00** |
| *Candidatus_Solibacter* | 13.91±1.87a | 14.09±1.66a | 16.08±2.22a | 15.64±1.59a | 16.33±2.06a | 15.83±1.27a | 18.83±2.01a | 0.58 |
| *Streptophyta* | 10.45±1.70ab | 19.91±5.68cd | 8.33±2.17a | 12.36±1.67ac | 17.08±3.23bc | 10.00±2.33a | 18.92±3.29bc | **0.05** |
| *Gp5* | 16.00±1.94ab | 20.55±2.78a | 17.00±3.00ab | 13.45±1.27b | 13.58±2.25b | 16.17±2.47ab | 12.67±2.17b | 0.26 |
| *Bacillus* | 9.91±1.96ab | 12.00±0.97ac | 8.75±1.28a | 12.73±1.14ac | 16.17±1.92c | 14.50±2.23bc | 10.42±1.45ab | **0.02** |
| *Rhizomicrobium* | 8.27±1.38a | 11.73±1.76ab | 13.00±1.61b | 13.64±1.87b | 10.25±1.62ab | 10.92±1.34ab | 13.08±1.36b | 0.21 |
| *Aciditerrimonas* | 13.36±1.53a | 9.27±1.21b | 9.25±1.27b | 9.55±1.33b | 10.83±1.02ab | 9.92±0.94ab | 10.75±1.55ab | 0.29 |

Different letters indicate statistical differences at a *P* value of < 0.05 (ANOVA) among rhizoma peanut cultivars by least-significant-difference (LSD) tests.
